# Supplementary material for: MAIT cells contribute to protection against lethal influenza infection in vivo
Source: Nat Commun. 2018 Nov 9;9:4706. doi: 10.1038/s41467-018-07207-9 (PMC6226485; doi:10.1038/s41467-018-07207-9)
Supplement: Supplementary file 1 — Supplementary Information [file 41467_2018_7207_MOESM1_ESM.pdf]

Supplementary information for:

MAIT cells contribute to protection against lethal influenza infection *in vivo*

by **van Wilgenburg et al**

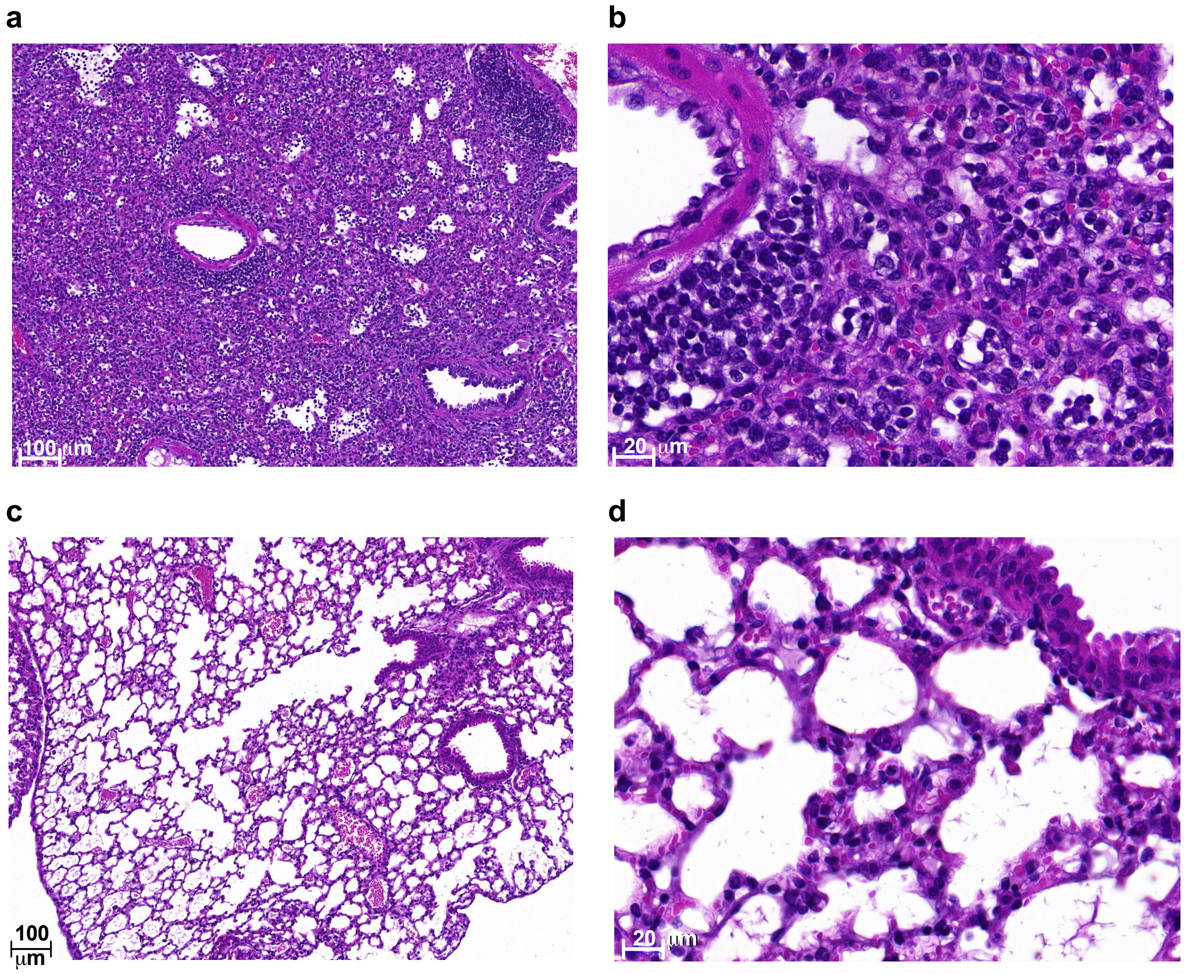

Supplementary Figure 1. Pulmonary histology during influenza virus infection.

Representative photomicrographs of haematoxylin and eosin-stained sections of lungs from C57BL/6 mice infected with 100 PFU of PR8 at 8 dpi. Images at low (**a**) and high (**b**) magnification show expansile parenchymal necrosis with severe perivascular, peribronchial and interstitial inflammation characterised by infiltrates of macrophages, lymphocytes and neutrophils. (**c, d**) Uninfected controls showing normal histology.

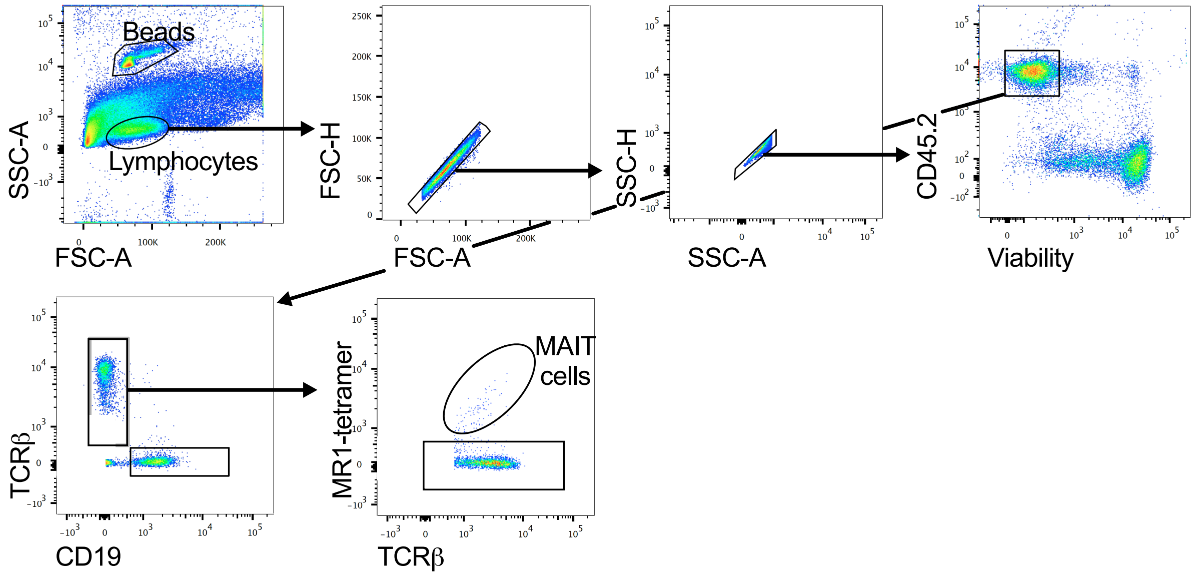

Supplementary Figure 2. Gating strategy used for MAIT cells.

Murine pulmonary MAIT cells are identified by gating on the lymphocyte population and excluding doublets using forward scatter / side scatter properties; dead cells are excluded using Fixable Aqua or Zombie Yellow; CD45.2+ live lymphocytes are gated and further selected as CD19- TCR-β+; TCR-β+ T cells or TCR-β+MR1-5-OP-RU-BV421 tetramer+ MAIT cells are then gated as shown. To derive absolute cell counts calibration beads were added prior to cytometry and identified by forward scatter / side scatter properties.

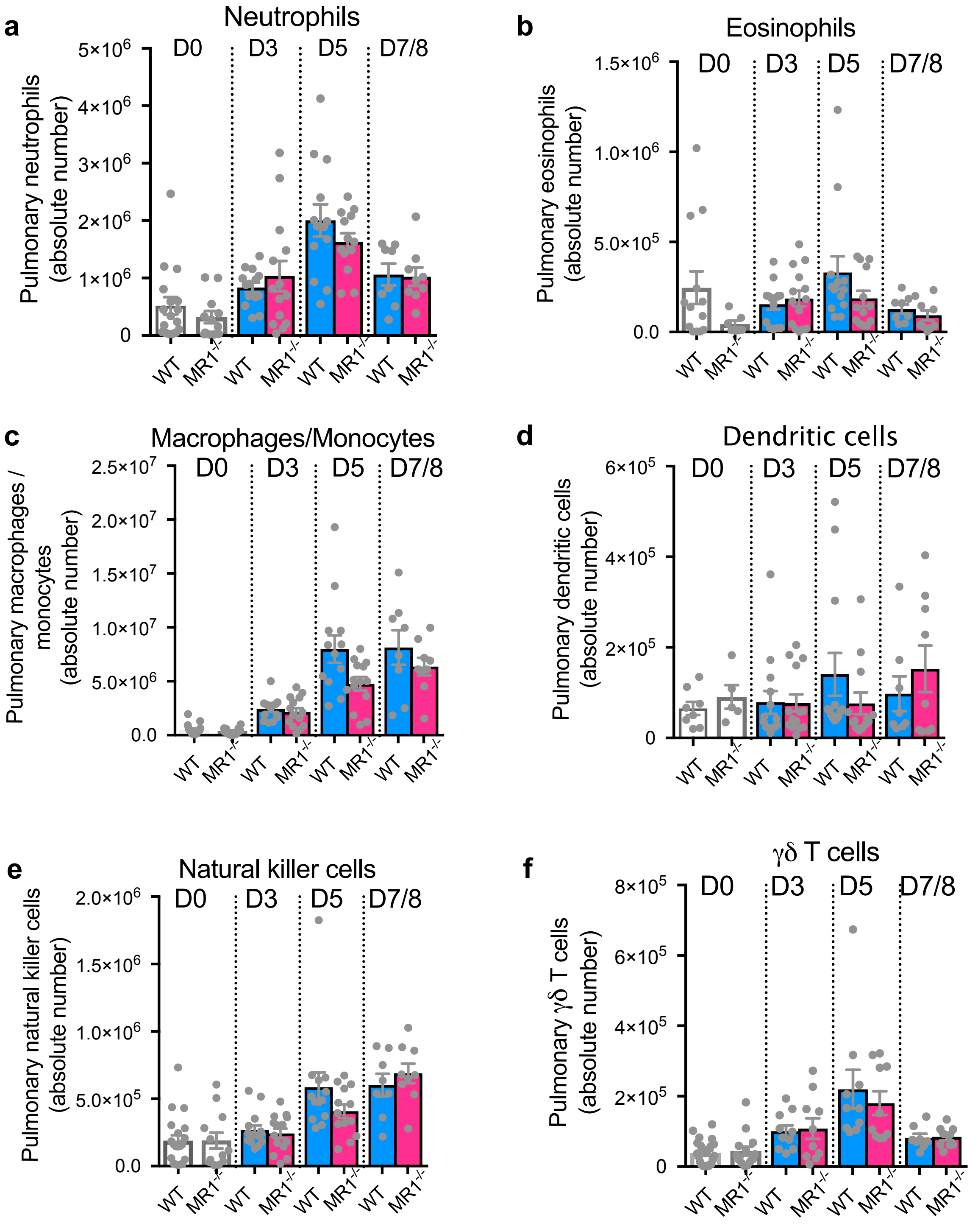

Supplementary Figure 3. Innate immune cell recruitment in wild-type and MR1$^{-/-}$ mice during PR8 infection.

(**a-f**) Absolute numbers of innate immune cells in the lungs of WT (C57BL/6) or MR1$^{-/-}$ mice before (D0) and at 3, 5 and 7 to 8 dpi (D3,D5,D7/8) following infection with 100-150 PFU of PR8. (**a**) neutrophils (Ly6G^{+}SigF^{-}CD11b^{+}), (**b**) eosinophils (SigF^{+}CD11b^{int}CD64^{-}CD11c^{-}), (**c**) macrophages/monocytes (CD64^{+}CD11b$^{+/-}$Ly6C$^{+/-}$SigF^{-}), (**d**) dendritic cells (CD11c^{+}I-A^{b+}CD3^{-}CD64^{-}, (**e**) natural killer cells (NK1.1^{+}CD3^{-}CD19^{-}CD11b^{-}F4/80^{-}CD11c^{-}), (**f**) γδ T cells (TCRγδ^{+}CD3^{+}TCRβ^{-}CD19^{-}CD11b^{-}F4/80^{-}CD11c^{-}). Graphs shows means ±SEM. Combined data from two experiments with similar results, (total n=8-14 per group).

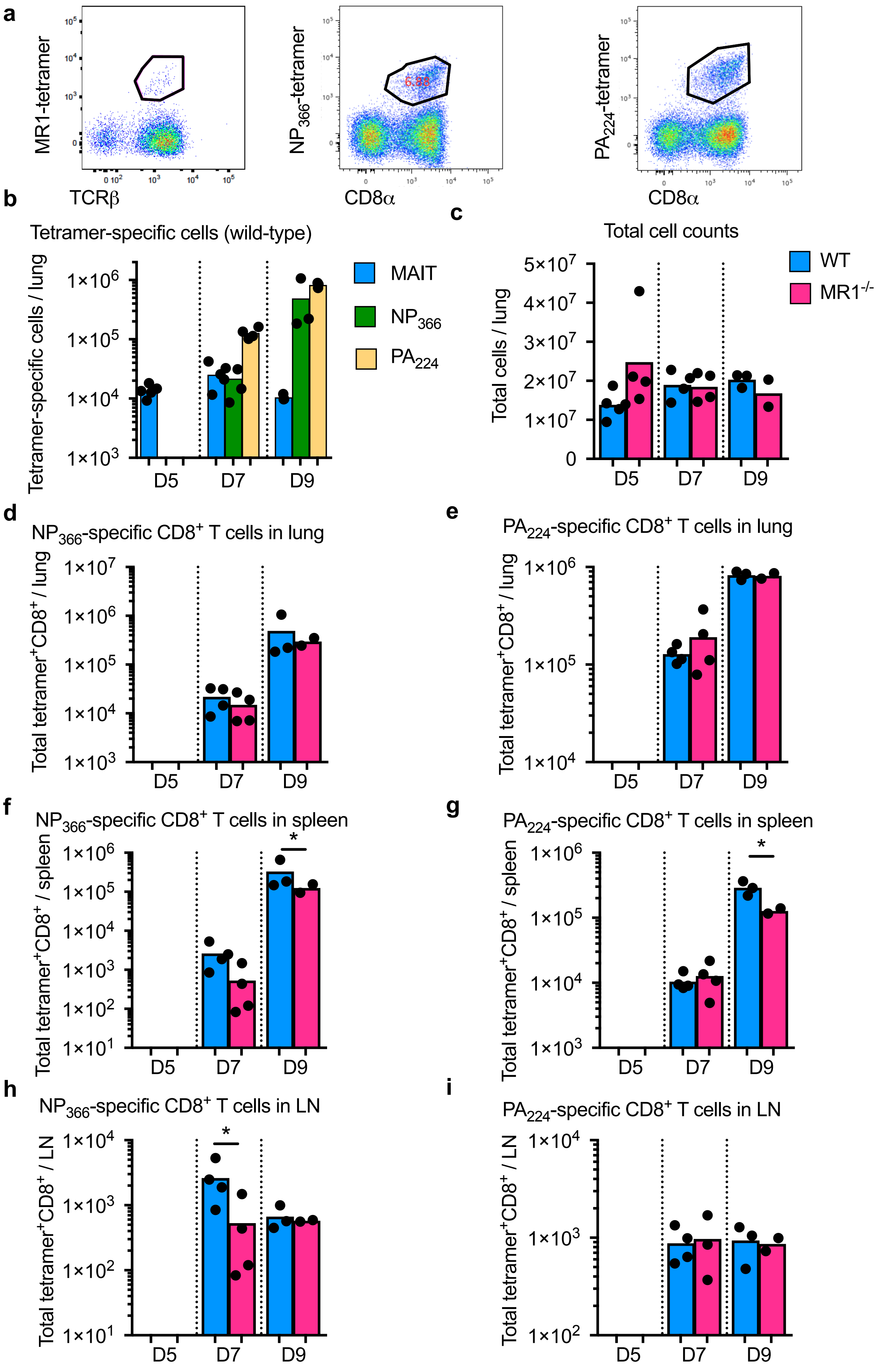
a
MR1-tetramer
TCRβ
NP366-tetramer
CD8α
PA224-tetramer
CD8α
b
Tetramer-specific cells (wild-type)
Tetramer-specific cells / lung
MAIT
NP366
PA224
D5
D7
D9
c
Total cell counts
Total cells / lung
WT
MR1-/-
D5
D7
D9
d
NP366-specific CD8+ T cells in lung
Total tetramer+CD8+ / lung
e
PA224-specific CD8+ T cells in lung
Total tetramer+CD8+ / lung
f
NP366-specific CD8+ T cells in spleen
Total tetramer+CD8+ / spleen
g
PA224-specific CD8+ T cells in spleen
Total tetramer+CD8+ / spleen
h
NP366-specific CD8+ T cells in LN
Total tetramer+CD8+ / LN
i
PA224-specific CD8+ T cells in LN
Total tetramer+CD8+ / LN

Supplementary Figure 4. Kinetics of tissue accumulation of MAIT cells and antigen-specific conventional CD8$^+$ T cells.

(**a**) Representative flow cytometry plots showing TCRβ and MR1-5-OP-RU tetramer staining (left), CD8α and nuclear protein (NP_{366}) tetramer (middle), and CD8α and polymerase acidic protein (PA_{224}) tetramer (right), gated on live, pulmonary CD45$^+$19$^-$ lymphocytes. (**b**) Changes in absolute numbers of MAIT cells and of conventional antigen-specific CD8α+ T cells specific for NP_{366} and PA_{224} in the lungs of wild-type mice 5, 7 and 9 days after intranasal infection with 100 PFU of PR8 virus. (**c-i**) Absolute numbers of all live cells (c) and of antigen-specific conventional T cells (d-i) in the lungs (c-e), spleen (f,g) and mediastinal lymph nodes (h,i) of wild-type and MR1-/- mice 5, 7 and 9 days after intranasal infection with 100 PFU of PR8 virus. Experiment performed once in 4-5 (day 5, 7) or 2-3 (day 9) surviving mice. Influenza-specific T cells were undetectable at day 5. *, $P<0.05$ using t-tests on log-transformed data.

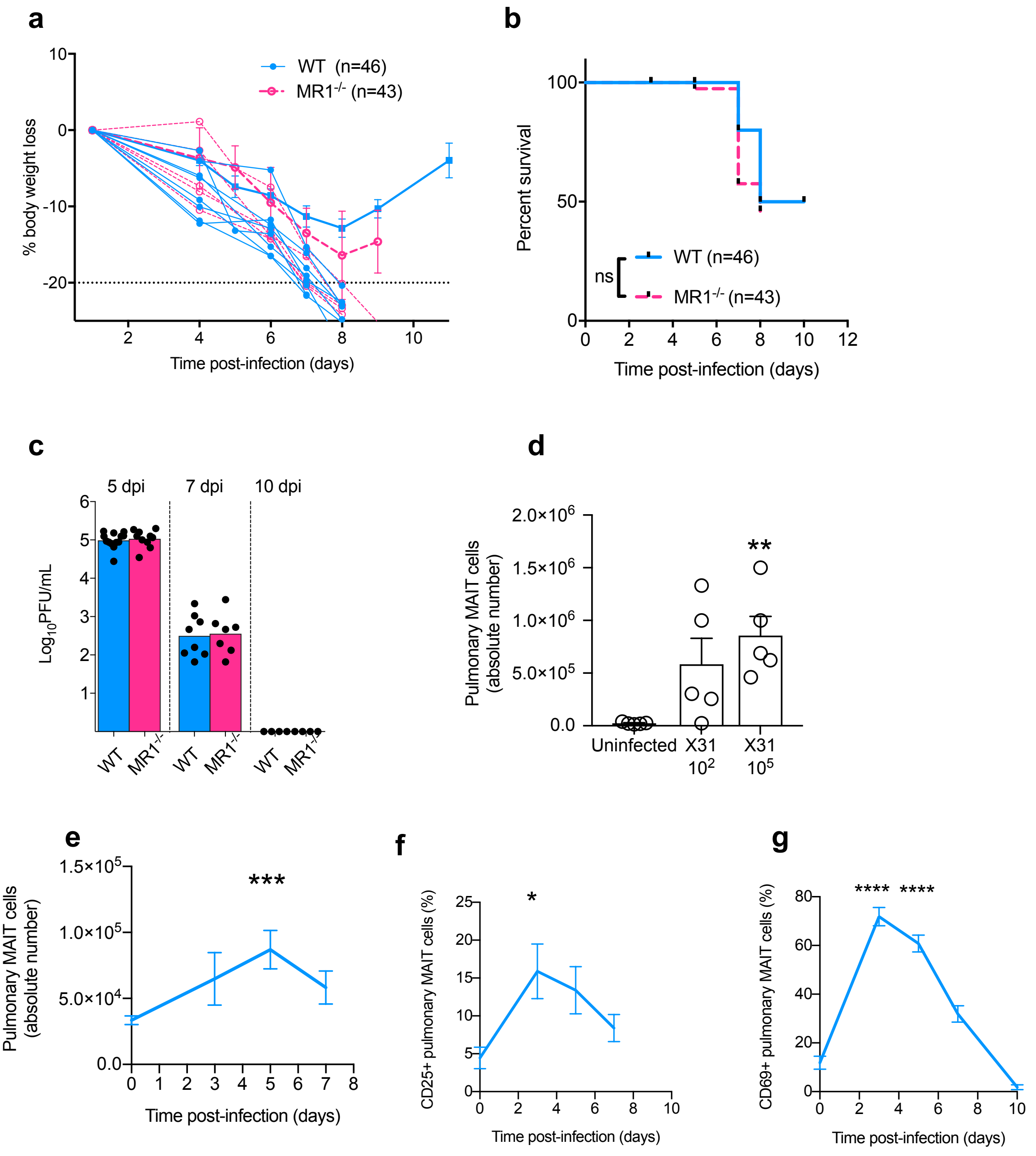

Supplementary Figure 5. MAIT cell activation and accumulation with the less pathogenic X-31 strain.

(**a**) Body weight loss expressed as a percentage of starting weight and (**b**) survival curves after infection with 5000 PFU of X-31 virus. Data are combined from five experiments with similar results, comprising WT (n=46) and $MR1^{-/-}$ (n=43) mice. Weight loss graphs show mean

weights±SEM for surviving mice, with individual plots for those which succumbed to infection. (**c**) Titres of infectious virus in clarified lung homogenates, expressed as PFU per lung at 5, 7 and 10 dpi. Data are from two independent experiments, each of n=3-6 per group. (**d**) Changes in absolute pulmonary MAIT cell numbers 7 days after intranasal infection with 100 or 10,000 PFU of X-31. Experiment performed once. (**e-g**) Absolute numbers of pulmonary MAIT cells (**e**) and proportions of MAIT cells expressing (**f**) CD25 and (**g**) CD69 in single-cell suspensions prepared from lungs before (D0) and after infection with 5000 PFU of X-31. Data are combined from three experiments with similar results. Graphs show means ±SEM. *, Mann-Whitney $P<0.05$.

a

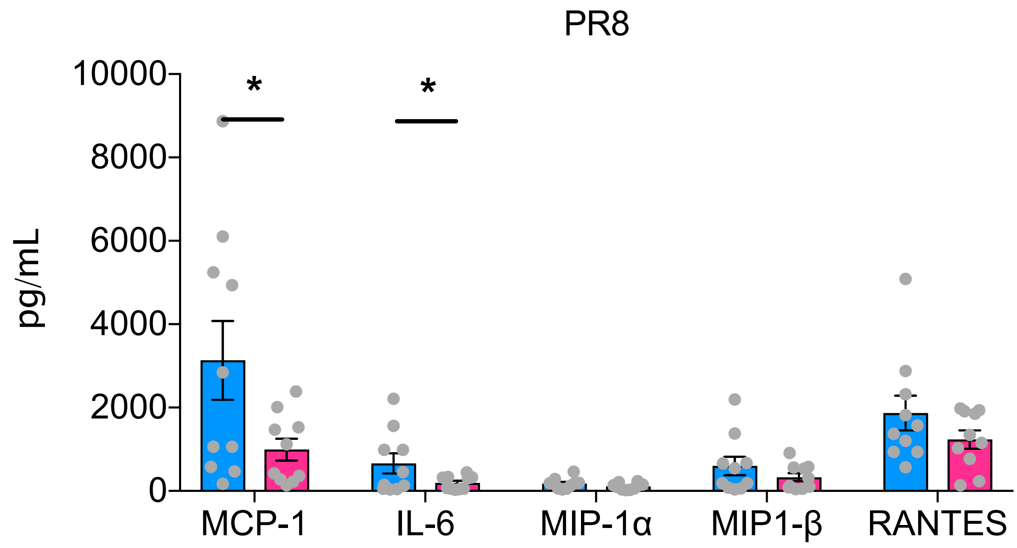

b

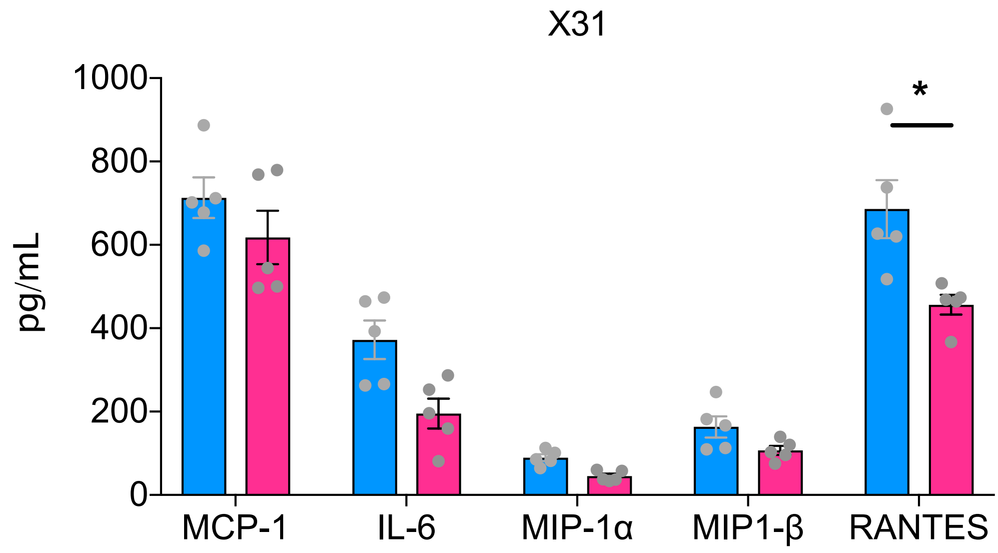

Supplementary Figure 6. Innate inflammatory cytokines in wild-type and MR1$^{-/-}$ mice.

Concentrations of key pro-inflammatory cytokines measured by cytokine bead array in homogenised lungs day 3 post-infection with (**a**) 100 PFU of PR8 and (**b**) 5000 PFU of X-31 compared in WT (C57BL/6) and MR1$^{-/-}$ mice. Mann-Whitney tests with Bonferroni correction

for multiple comparisons. Graphs show means ±SEM of n=10 (PR8) or 5 (X-31) mice. *, $P<0.05$; **, $P<0.001$.

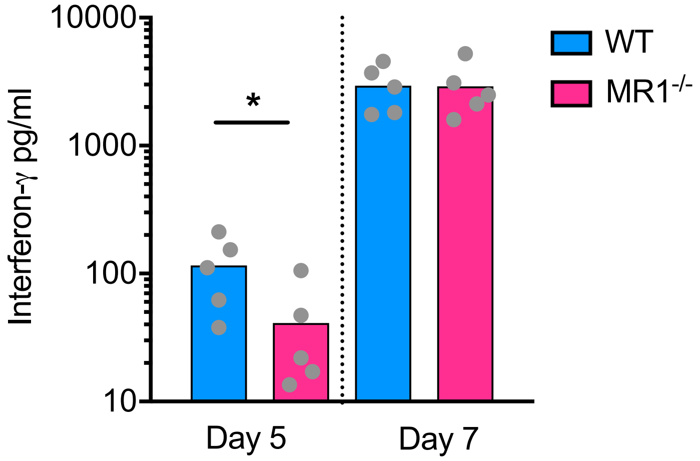

Supplementary Figure 7. Interferon-γ in bronchoalveolar lavage fluid from wild-type and MR1$^{-/-}$ mice.

Concentrations of interferon-γ measured by cytokine bead array in bronchoalveolar lavage fluid on days 5 and 7 post-infection with 100 PFU of PR8 virus compared in WT (C57BL/6) and MR1$^{-/-}$ mice. Experiment performed once. *, P<0.05 with t-test on log-transformed data. Graphs show means of n=5 mice per group.

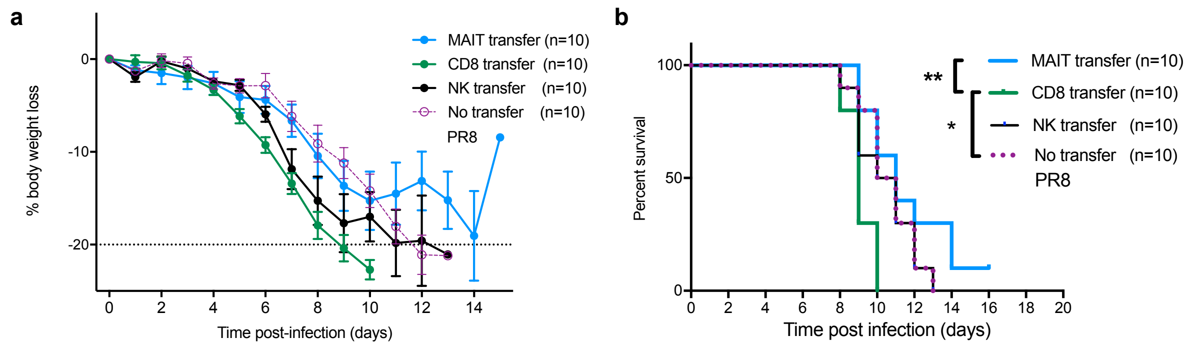

Supplementary Figure 8. Comparative effect of adoptive transfer of MAIT cell, NK cells and CD8 cells in Rag2$^{-/-}\gamma$C$^{-/-}$ mice.

1 x 10^5 pulmonary MAIT (CD3$^+$CD45.2$^+$TCRβ$^+$MR1-5-OP-RU tetramer$^+$), NK (CD45.2$^+$CD8α$^-$CD11b$^-$CD11c$^-$B220$^-$F4/80$^-$NK1.1$^+$) or CD8 (TCRβ+ CD45.2$^+$CD11b$^-$ CD11c$^-$B220$^-$F4/80$^-$CD8$^+$) cells from C57BL/6 mice (previously infected with 10^6 CFU *S.* Typhimurium BRD509 for 7 days to expand the MAIT cell population) were sorted and transferred intravenously into Rag2$^{-/-}\gamma$C$^{-/-}$ mice, followed by intraperitoneal anti-CD4 and anti-CD8 antibody injection (0.1 mg each) twice within 1 week to deplete any residual conventional T cells included in the transfer. After 2 weeks, mice were infected i.n. with 25 PFU of PR8. For schematic see Fig 4a. (**a**) Body weight loss expressed as a percentage (showing mean±SEM), and (**b**) survival curves compared using log-rank (Mantel-Cox) tests (P=0.004) and Grehan-Breslow-Wilcoxon tests (P=0.012) between groups. * P<0.05, ** P<0.01 denote significant differences in *post hoc* comparisons between individual groups using log-rank tests with Bonferroni correction. Other differences were not significant. Data represent combined data from two experiments with similar results, each using 5 mice per group, per replicate.
