# Supplementary material for: MAIT cells contribute to protection against lethal influenza infection in vivo
Source: Nat Commun. 2018 Nov 9;9:4706. doi: 10.1038/s41467-018-07207-9 (PMC6226485; doi:10.1038/s41467-018-07207-9)
Supplement: Supplementary file 3 — Reporting Summary [file 41467_2018_7207_MOESM3_ESM.pdf]

nature research

Corresponding author(s): Paul Klenerman

Reporting Summary

Nature Research wishes to improve the reproducibility of the work that we publish. This form provides structure for consistency and transparency in reporting. For further information on Nature Research policies, see Authors & Referees and the Editorial Policy Checklist.

Statistical parameters

When statistical analyses are reported, confirm that the following items are present in the relevant location (e.g. figure legend, table legend, main text, or Methods section).

n/a	Confirmed	
☐	☒	The exact sample size (*n*) for each experimental group/condition, given as a discrete number and unit of measurement
☐	☒	An indication of whether measurements were taken from distinct samples or whether the same sample was measured repeatedly
☐	☒	The statistical test(s) used AND whether they are one- or two-sided<br>*Only common tests should be described solely by name; describe more complex techniques in the Methods section.*
☒	☐	A description of all covariates tested
☐	☒	A description of any assumptions or corrections, such as tests of normality and adjustment for multiple comparisons
☐	☒	A full description of the statistics including central tendency (e.g. means) or other basic estimates (e.g. regression coefficient) AND variation (e.g. standard deviation) or associated estimates of uncertainty (e.g. confidence intervals)
☐	☒	For null hypothesis testing, the test statistic (e.g. *F*, *t*, *r*) with confidence intervals, effect sizes, degrees of freedom and *P* value noted<br>*Give P values as exact values whenever suitable.*
☒	☐	For Bayesian analysis, information on the choice of priors and Markov chain Monte Carlo settings
☒	☐	For hierarchical and complex designs, identification of the appropriate level for tests and full reporting of outcomes
☒	☐	Estimates of effect sizes (e.g. Cohen's *d*, Pearson's *r*), indicating how they were calculated
☐	☒	Clearly defined error bars<br>*State explicitly what error bars represent (e.g. SD, SE, CI)*

Our web collection on statistics for biologists may be useful.

Software and code

Policy information about availability of computer code

Data collection	Cytometry data were collected with DIVA v7 BD Biosciences
Data analysis	Prism GraphPad software (version 7.0 La Jolla, CA). FlowJo10 software (Ashland, OR)

For manuscripts utilizing custom algorithms or software that are central to the research but not yet described in published literature, software must be made available to editors/reviewers upon request. We strongly encourage code deposition in a community repository (e.g. GitHub). See the Nature Research guidelines for submitting code & software for further information.

Data

Policy information about availability of data

All manuscripts must include a data availability statement. This statement should provide the following information, where applicable:

- Accession codes, unique identifiers, or web links for publicly available datasets
- A list of figures that have associated raw data
- A description of any restrictions on data availability

Data supporting the findings of this study are available within the paper and its supplementary information files, or are available from the authors upon request.

Field-specific reporting

Please select the best fit for your research. If you are not sure, read the appropriate sections before making your selection.

☒ Life sciences ☐ Behavioural & social sciences ☐ Ecological, evolutionary & environmental sciences

For a reference copy of the document with all sections, see nature.com/authors/policies/ReportingSummary-flat.pdf

Life sciences study design

All studies must disclose on these points even when the disclosure is negative.

Sample size	Sample sizes were estimated based on previous extensive experience in the laboratory with these PR8 and X-31 strains.
Data exclusions	No data were excluded from the analyses
Replication	Numbers experimental replicates are stated in each figure legend. No data are presented where discrepant findings were observed between replicates. In most instances (except where stated) significant findings were performed at least twice and up to four times, as stated in each relevant figure legend.
Randomization	Randomisation was not performed, but all groups being compared were experimented on in parallel in a single batch with a single inoculation and using weight and gender matching, with housing, feeding and husbandry matched to minimise covariates.
Blinding	Blinding was not possible during cytometry experiments as MAIT cells are absent from MR1-/- mice, and thus the species phenotype could not be concealed from the operator.

Reporting for specific materials, systems and methods

Materials & experimental systems

n/a	Involved in the study
☒	☐ Unique biological materials
☐	☒ Antibodies
☒	☐ Eukaryotic cell lines
☒	☐ Palaeontology
☐	☒ Animals and other organisms
☒	☐ Human research participants

Methods

n/a	Involved in the study
☒	☐ ChIP-seq
☐	☒ Flow cytometry
☒	☐ MRI-based neuroimaging

Antibodies

Antibodies used	MR1-5-OP-RU-SA BV421 tetramers were generated as described previously9 (now available from the NIH core tetramer facility) and influenza-specific MHC Class I tetramers targeting immunodominant epitopes of nucleoprotein (DbNP366) and polymerase acidic protein (DbPA224) and were utilised conjugated to SA-APC and PE respectively. Antibodies against murine CD3e (clone 145-2C11, catalogue number 561108, fluorochrome PerCP-Cy5.5, dilution 1:200), CD4 (GK1.5, 552051, APC-Cy7, 1:200), CD11b (M1/70, 563015, BV605, 1:600), CD19 (1D3, 561113, PerCP-Cy 5.5), CD45.2 (104, 553772, FITC and 563685, BV711, 1:400), CD69 (H1.2F3, 12-0691-81, PE and 70318, FITC), IFN-? (XMG1.2, 557649, PE-Cy7), Ly6C (AL-21, 561237, AF700, 1:400), Ly6G (IA8, 560601, PE-Cy7, 1:1000), NK1.1 (PK136, 553165, PE, 1:200), SiglecF (E50-2440, 562757, PECF594, 1:1000), TCR? (H57-597, 553174, APC or 553172, PE, 1:500) were purchased from BD (Franklin Lakes, NJ). Antibodies against CD3 (17A2, 56-0032-80, AF700, 1:100) CD8? (53-6.7, 12-0081-81, PE), CD11c (N418, 17-01114-81, FITC, 1:1000), CD25 (PC61.5, 17-02510-81, APC), F4/80 (BM8, 11-4801-81, FITC, 1:100), Granzyme B (NGZB, 12-8898-80, PE) were purchased from eBioscience (San Diego, CA). Abs against CD19 (6D5, 115546, BV510), F4/80 (BM8, 123116, APC), CD11b (M1/70, 101206, FITC, 1:1000), CD11c (N418, 117336, BV786, 1:400), CD19 (6D5, 115506, FITC, 1:500), CD27 (LG.3A10, 124214, PerCP-Cy5.5, 1:400), CD62L (Mel-14, 104406, FITC, 1:800 and PE-Cy7, 1:500), CD64 (X54-5/71, 139322, AF647, 139311, BV711, 1:400, I-Ab, AF6-120.1, 116422, Pacific Blue, 1:400) were purchased from Biolegend (San Diego, CA). To block non-specific staining, cells were incubated with unlabelled MR1-6-formylpterin tetramer and anti-Fc receptor (2.4G2) for 15 min at room temperature and then incubated at room temperature with Ab/tetramer cocktails in PBS/2% foetal calf serum. 7-aminoactinomycin D (5 ?l per sample) was added for the last 10 min or fixable viability stains added according to the manufacturer's instructions: Live/Dead Fixable Aqua (L34957, ThermoFisher, Waltham, MA, 1:800) and ZombieYellow (423104, Biolegend).
Validation	Antibody validation for the species - mice - is provided for all commercially available antibodies described above on the relevant manufacturer's website accessible via the catalogue numbers which are also provided above. For the tetramers validation is

provided in the previous literature from our group, specifically the following publication which is cited in the manuscript:Corbett, A.J., et al. T-cell activation by transitory neo-antigens derived from distinct microbial pathways. Nature 509, 361-365 (2014).

Animals and other organisms

Policy information about studies involving animals; ARRIVE guidelines recommended for reporting animal research

Laboratory animals	Mice were bred and housed in the Biological Research Facility of the Peter Doherty Institute (Melbourne, Victoria, Australia). MR1-/- mice were generated by breeding Va19iCa-/-MR1-/- mice47 (from Susan Gilfillan, Washington University, St Louis School of Medicine, St Louis, MO) with C57BL/6 mice and inter-crossing of F1 mice. The genotype was determined by tail DNA PCR at the MR1 locus using the following primers29: Fwd: AGC TGA AGT CTT TCC AGA TCG; Rev (wild type): ACA GTC ACA CCT GAG TGG TTG; Rev (knockout): GAT TCT GTG AAC CCT TGC TTC. IFN-? knockout mice (B6.129S7-Ifngtm1Ts/J)48 are commercially available (Jackson Laboratory). Male mice aged 6–12 weeks, matched for age, sex and weight, without randomisation or blinding, were used.
Wild animals	N/A
Field-collected samples	N/A

Flow Cytometry

Plots

Confirm that:

☒ The axis labels state the marker and fluorochrome used (e.g. CD4-FITC).

☒ The axis scales are clearly visible. Include numbers along axes only for bottom left plot of group (a 'group' is an analysis of identical markers).

☒ All plots are contour plots with outliers or pseudocolor plots.

☒ A numerical value for number of cells or percentage (with statistics) is provided.

Methodology

Sample preparation	Mice were killed by CO2 asphyxia, the heart perfused with 10 ml cold RPMI and lungs were taken. To prepare single-cell suspensions lungs were finely chopped with a scalpel blade and treated with 3 mg ml-1 collagenase III (Worthington, Lakewood, NJ), 5 ?g ml-1 DNAse, and 2% foetal calf serum in RPMI for 90 min at 37?C with gentle shaking. Cells were then filtered (70 ?m) and washed with PBS/2% foetal calf serum. For plaque assays lungs were placed into RPMI and homogenised using a Polytron System PT 1200 CL 230V (Kinematica, Lucerne, Switzerland). Red blood cells were lysed with hypotonic buffer TAC (Tris-based amino chloride) for 5 min at 37?C. Approximately 1.5x106 cells were filtered (40 ?m) and used for flow cytometric analysis. Absolute cell counts were derived by adding to each sample 2.5x104 blank calibration particles (BD Pharmingen).
Instrument	Data were collected on LSRII or LSR Fortessa or Canto II (BD Biosciences) flow cytometers.
Software	Software was BD DIVA version 7
Cell population abundance	For adoptive transfer MAIT frequencies post salmonella were typically 30-50% of all pulmonary T cells. Sort purity was >=98% and was checked by passing the post sort samples through the Aria again.
Gating strategy	Murine pulmonary MAIT cells are identified by gating on the lymphocyte population and excluding doublets using forward scatter / side scatter properties; dead cells are excluded using Fixable Aqua or Zombie Yellow; CD45.2+ live lymphocytes are gated and further selected as CD19- TCR-?+; TCR-?+ T cells or TCR-?+MR1-5-OP-RU-BV421 tetramer+ MAIT cells are then gated as shown. To derive absolute cell counts calibration beads were added prior to cytometry and identified by forward scatter / side scatter properties. The boundaries are described in the gating figure Suplpementary Figure 2

☒ Tick this box to confirm that a figure exemplifying the gating strategy is provided in the Supplementary Information.
